# Influence of the Structural Properties of Mesoporous Silica on the Adsorption of Guest Molecules

**DOI:** 10.3390/ma3084500

**Published:** 2010-08-25

**Authors:** Hanna Ritter, Jan Hinrich Ramm, Dominik Brühwiler

**Affiliations:** Institute of Inorganic Chemistry, University of Zurich, Winterthurerstrasse 190, CH-8057 Zurich, Switzerland

**Keywords:** mesoporous silica, host-guest, adsorption, interaction, amine, fluorescein, FITC

## Abstract

Amino-functionalized mesoporous silica of different pore sizes and pore system dimensionalities is used as a host material for the inclusion of fluorescein (non-covalent host-guest interaction) and fluorescein isothiocyanate (covalent host-guest interaction). The parameters determining the achievable guest loading depend on the type of host-guest interaction. For covalent interaction, the loading is mainly determined by the accessibility of the adsorption sites, while a more complex situation was encountered in case of non-covalent interactions. In addition to the accessibility of the adsorption sites, an interpretation of the results needs to take into account the confinement of the included guests, as well as the distribution of the adsorption sites.

## 1. Introduction

The introduction of guests into nanoporous hosts with defined pore sizes is a versatile concept for producing materials with new properties and promising possibilities for various applications [1,2,3,4]. Examples include photonic antenna systems (host = zeolite L, guest = luminescent molecules) [4], drug delivery devices (host = mesoporous silica, guest = drug molecules) [5,6,7], and biocatalysts (host = mesoporous silica, guest = enzymes) [8]. The discovery of mesoporous silica with ordered pores [9,10] has extended this field by allowing the incorporation of large guest species such as DNA [11], preformed quantum dots [12], or semiconducting polymers [13]. When preparing host-guest materials of this kind, questions regarding the ideal pore size and pore system arise. Using a model consisting of fluorescein and fluorescein isothiocyanate (FITC) as guests and various amino-functionalized mesoporous silica materials as hosts, we illustrate that the answer to these questions very much depends on the particular host-guest interaction.

The pore size of a host material determines the accessibility of the adsorption sites. It is intuitively immediately clear that the accessibility increases with increasing pore size, thereby enabling the preparation of highly loaded host-guest materials. However, large pore sizes usually promote leaching, particularly in cases where the interaction between guest and host is weak. This leads to experimental problems, because the preparation of host-guest systems based on nanoporous materials usually includes washing steps to remove guest species adsorbed on the external particle surface. In case of a non-covalent interaction between guest and host, it is therefore necessary to optimize the accessibility of the adsorption sites without compromising the confinement. Confinement can be a crucial factor in determining the stability of host-guest systems, as shown by the inclusion of organic molecules into the narrow channels of zeolite L, where it has been observed that leaching can be suppressed under certain conditions, even when washing with a solvent in which the guests are well soluble [14].

When working with covalent host-guest interactions, leaching of the guests becomes much less of a problem. However, in terms of the synthesis of the respective host-guest systems, pore blocking effects by irreversible binding of guests to pore entrance sites have to be taken into account when choosing an appropriate host material.

## 2. Results and Discussion

### 2.1. Mesoporous Silica

**Figure 1:**
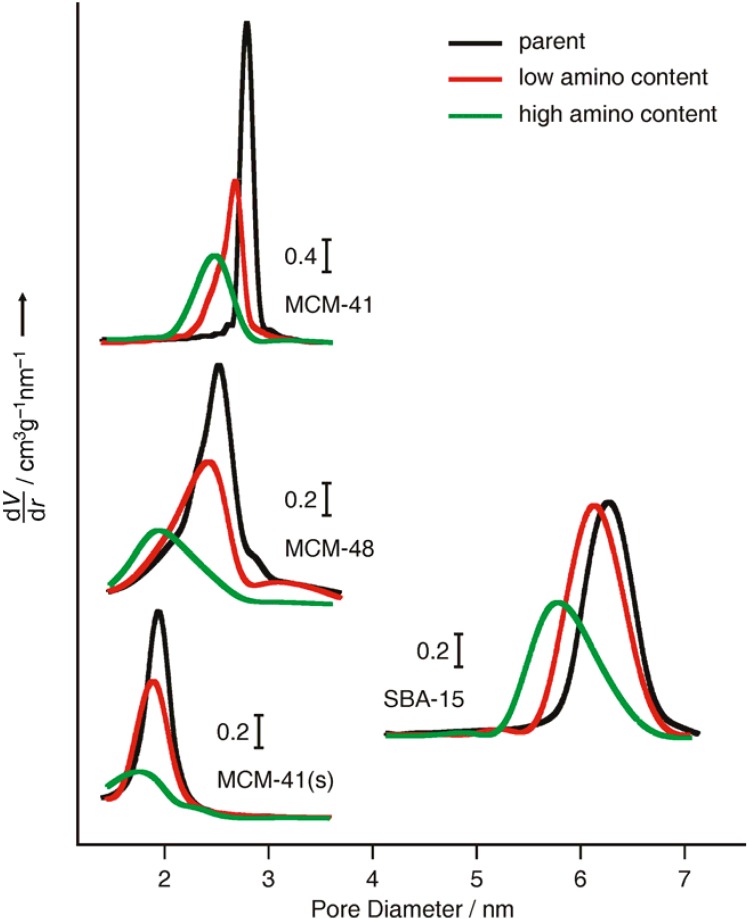
Pore size distributions (BJH, desorption isotherm) of parent and amino-functionalized mesoporous silicas.

To study the effect of the pore size on the ability of the host materials to accommodate fluorescein and FITC, we have employed various mesoporous silicas. The key characteristics of the parent materials (before amino-functionalization), namely the pore diameter (d_BJH_), the BET surface area (S_BET_), the external particle surface area (S_Ext_), the total pore volume (V_tot_), and the primary mesopore volume (V_P_), are summarized in Table 1. Pore size distributions are given in Figure 1. It should be noted that the BJH method tends to underestimate the absolute pore size [15]. This is illustrated by comparison with values obtained using the geometrical method proposed by Kruk *et al.*, which additionally takes into account the X-ray diffraction data and yields reliable results for well defined MCM-41 type systems [16]. Applying this method resulted in a pore diameter of 2.89 nm for MCM-41(s) and 3.76 nm for MCM-41. All materials feature particles of irregular morphology with particle sizes in the range of 1-2 µm.

**Figure 2:**
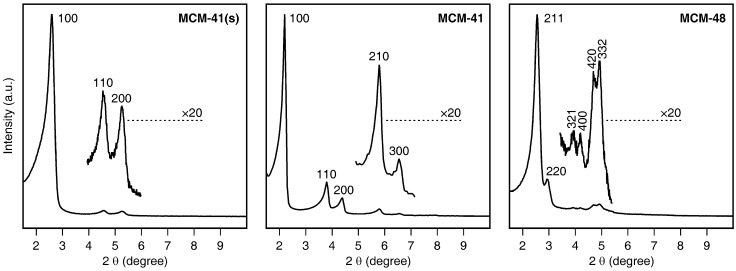
X-ray diffraction patterns of parent MCM-41(s) (left panel), MCM-41(middle panel), and MCM-48(right panel).

Apart from the pore sizes, there are further fundamental differences between the materials. While MCM-41(s), MCM-41, and SBA-15 have one-dimensional channel systems (hexagonal), the pore system of MCM-48 is three-dimensional (cubic) (Figure 2) [10]. SBA-15, on the other hand, has a property that is commonly not found in materials of the MCM-41 and MCM-48 type. The pore walls of SBA-15 contain micropores, depending to a certain extent on the synthesis conditions [17]. In our case, a micropore volume of 0.09 cm^3^/g was found.

**Table 1:** Properties of the parent mesoporous silica materials.

	d_BJH_ [nm]	S_BET_ [m^2^/g]	S_Ext_ [m^2^/g]	V_tot_ [cm^3^/g]	V_P_ [cm^3^/g]
MCM-41(s)	1.96	776	35	0.48	0.44
MCM-48	2.52	1320	173	0.98	0.86
MCM-41	2.82	872	66	0.74	0.67
SBA-15	6.46	908	74	1.21	1.09

Amino-functionalized materials were obtained by the well established reaction with 3-aminopropyltrimethoxysilane (APTMS). Samples with low (approximately 0.45 µmol/m^2^) and high amino content (approximately 1.35 µmol/m^2^) were prepared by adding the corresponding amounts of APTMS to a suspension of the mesoporous silica in dry toluene. Indeed, analysis of the amino-functionalized samples revealed that grafting of APTMS was close to quantitative under our conditions, and led, as expected, to a reduction of the average pore diameter, pore volume, and BET surface area of the materials. The respective pore size distributions are shown in Figure 1.

### 2.2. Loading with FITC and Fluorescein

**Figure 3:**
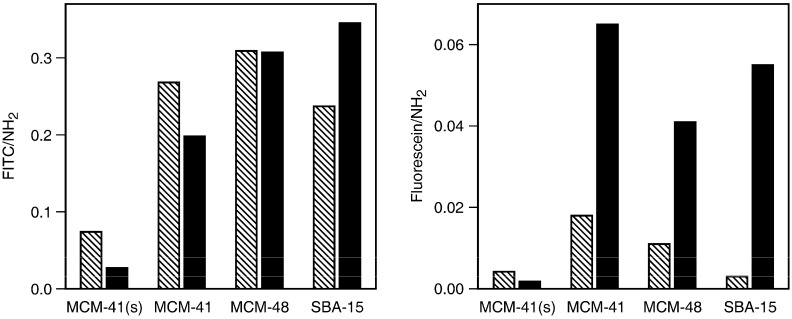
Amount of included FITC (left) and fluorescein (right) relative to the amount of surface-grafted amino groups for low (hatched bars) and high (black bars) amino contents.

**Figure 4:**
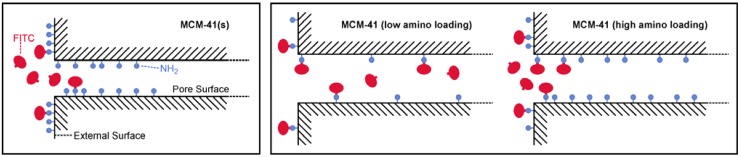
Schematic snapshots illustrating the reaction of FITC with amino-functionalized MCM-41(s) and MCM-41.

FITC is a popular amine labeling reagent, forming a robust thiourea upon reaction. In combination with mesoporous silica, FITC has been used to visualize the distribution of surface-grafted amino groups [18,19,20], as well as to prepare materials with pH sensing [21] and bioimaging capabilities [22,23,24,25]. Independent loading experiments with the same amino-functionalized sample gave FITC contents that were typically within ±10 %. The interpretation of the FITC loading experiments (Figure 3, left) is rather straightforward and can be reduced to an assessment of the accessibility of the amino groups [26]. Having the smallest pore size, MCM-41(s) is able to bind only small amounts of FITC. This is most likely due to pore blocking upon reaction of FITC with amino groups located close to the pore entrances (Figure 4). It should be noted in this context that the distribution of the grafted amino groups is most likely non-uniform, especially in materials with small mesopores, leading to higher grafting densities near the pore entrances [27,28,29], and thus further promoting pore blocking upon reaction with FITC. Such effects are less pronounced in MCM-41 as a consequence of the larger pores, leading to increased amounts of FITC-labeled amino groups. The relative amount of coupled FITC decreases upon increasing the amino content, because the probability of FITC binding to pore entrance sites increases (Figure 4). Given the pore size of MCM-48, it is very likely that pore blocking occurs in a similar manner as observed for MCM-41. Due to its three-dimensional channel system, the effect of pore blocking on the accessibility of the amino groups, and therefore on the amount of coupled FITC, is, however, less dramatic. Following this line of reasoning, one would expect to find the largest amounts of bound FITC in the amino-functionalized SBA-15. While this is indeed the case for high amino loading, the SBA-15 sample containing small amounts of grafted amino groups gave a comparatively low amount of coupled FITC. This is in agreement with a previous study that led to the conclusion that APTMS grafts preferentially to the intrawall micropores, thereby becoming unavailable for reaction with FITC [26].

In the case of fluorescein, the results of the loading experiments (Figure 3, right) and their interpretation are more complex due to the reversible interaction of the guest molecules with the host. As expected, the amounts of included fluorescein are much smaller compared to the amounts of bound FITC. Nonetheless, we found the results to be well reproducible. Independent loading experiments with the same amino-functionalized sample gave fluorescein contents that were within ±10 %.

With pK_a_ values of 4.4 (neutral fluorescein) and 6.7 (fluorescein monoanion) [30], we can expect protonation of surface-grafted amino groups and subsequent electrostatic interaction with fluorescein mono- and dianions. Fluorescein contents clearly show a dependence on the pore size that is different to the one observed for FITC, thus indicating that in addition to the accessibility of the amino groups, there must be further parameters determining the amount of adsorbed fluorescein. MCM-41 in particular is able to retain an uncharacteristically large amount of fluorescein. This can be explained on the basis that fluorescein molecules adsorbed in the channels are well protected against washing with ethanol, whereas in the case of MCM-41(s) most of the fluorescein molecules are located on or close to the exposed external surface. Similarly, a large pore diameter (SBA-15) leads to less confinement, rendering the adsorbed fluorescein molecules susceptible to removal by washing. In this context, it is interesting to note that the dependence of the leaching rate on the pore diameter of a host can potentially be used to control the release of substances [31]. Regarding the microporosity of SBA-15, we can conclude that based on the amount of retained fluorescein, adsorption in the intrawall micropores, which would provide strong confinement, is unlikely. Again it is instructive to compare the performances of MCM-41 and MCM-48. The three-dimensional channel system apparently facilitates the removal of adsorbed fluorescein molecules during the washing process.

In contrast to the loading experiments with FITC, the amounts of included fluorescein (relative to the amount of amino groups) are considerably higher in case of high amino contents. To explain this significant difference between low and high amino contents, we have to consider that site-isolated amino groups are more likely at low amino contents. While such site-isolated amino groups can effectively bind FITC, they are less capable of adsorbing and retaining fluorescein. At high amino contents, on the other hand, one can expect a certain degree of APTMS cross-linking, leading to surface-anchored clusters of aminopropyl moieties [32]. Such patches of closely spaced amino groups can effectively adsorb and retain fluorescein.

## 3. Experimental Section

### 3.1. Synthesis of Mesoporous Silica

**MCM-41 and MCM-41(s)** [26]: 2.20 g of hexadecyltrimethylammonium bromide (CTAB, Fluka) was dissolved under slight warming (approx. 35 °C) in a mixture of 52 mL of H_2_O and 24 mL of aqueous ammonia (28 %, Fluka). An amount of 10 mL of tetraethoxysilane (TEOS, Fluka) was slowly added under stirring and the resulting gel was further stirred for 3 h at room temperature. The mixture was transferred to a Teflon-lined autoclave and heated at 110 °C for 48 h. The product was obtained by filtration, washed with at least 800 mL of H_2_O and dried overnight in air at room temperature. The structure directing agent (SDA) was removed by first heating at 300 °C for 2 h and subsequent calcination in air at 550 °C for 16 h. Heating rates of 2 °C/min were applied.

MCM-41 featuring a smaller pore diameter (MCM-41(s)) was prepared accordingly, using 1.86 g of dodecyltrimethylammonium bromide (Fluka) instead of CTAB.

**MCM-48** [33]: An amount of 8.80 g of CTAB was dissolved under slight warming (approx. 35 °C) in 80 mL of H_2_O. After the addition of 10 mL of 2 M aqueous NaOH, 10 mL of TEOS was added dropwise under stirring. After further stirring for 30 min, the mixture was transferred to a Teflon-lined autoclave and heated at 100 °C for 72 h. The product was recovered by filtration, washed with at least 1 L of H_2_O and oven-dried overnight at 80 °C. The SDA was removed by first heating at 300 °C for 2 h and subsequent calcination in air at 550 °C for 8 h. Heating rates of 2 °C/min were applied.

**SBA-15** [34]: 2.20 g of Pluronic P123 (EO_20_PO_70_EO_20_, M_av_ = 5800, Aldrich) was dissolved in a mixture of 49 mL of H_2_O and 31 mL of 4 M aqueous HCl. To this clear solution, 5 mL of TEOS was slowly added under stirring. After further stirring for 20 h at approximately 35 °C, the mixture was transferred to a Teflon-lined autoclave and heated at 100 °C for 24 h. The product was obtained by filtration and washed with at least 1 L of H_2_O. After drying the material overnight in air at room temperature, the SDA was removed by heating in air at 500 °C for 16 h, with a heating rate of 1 °C/min.

### 3.2. Reaction with APTMS

For the functionalization with 3-aminopropyltrimethoxysilane (APTMS, Fluka), 500 mg of calcined mesoporous silica was dispersed in 30 mL of dry toluene (Fluka, puriss.). After the addition of a calculated amount of APTMS (taking into account the surface area of the employed mesoporous silica and a quantitative grafting yield), the suspension was refluxed for 3 h. The functionalized product was recovered by filtration, washed with 100 mL of ethanol, and cured at 80 °C for 1 h.

### 3.3. Loading with FITC and Fluorescein

A calculated amount (1.5-fold excess relative to the amount of grafted amino groups) of FITC (fluorescein 5-isothiocyanate, Fluka) or fluorescein (free acid, Riedel-de Haën) was dissolved in 25 mL of absolute ethanol. After the addition of 250 mg of amino-functionalized mesoporous silica, the suspension was stirred for 24 h at room temperature. The yellow product was recovered by filtration and washed with 50 mL of ethanol. After redispersion in 50 mL of fresh ethanol and stirring for 15 min, the final product was recovered by filtration, washed with 50 mL of ethanol and oven-dried at 80 °C for 1 h.

The amount of included FITC or fluorescein was determined by dissolving the sample in 25 mL of 0.2 M aqueous NaOH and measuring the UV-Vis absorption spectrum of the resulting clear solution after appropriate dilution. Repeated analysis of the same sample gave an average relative error of 3 %. An extinction coefficient of ε = 75'000 M^–1^cm^–1^ at λ = 490 nm was used for calculating the concentration [26].

### 3.4. Amino Group Analysis

An amount of 15 mg of amino-functionalized mesoporous silica was stirred in 30 mL of 0.02 M aqueous NaOH until completely dissolved. A 100 µL aliquot of this solution was transferred into a cuvette (d = 1 cm) and 2 mL of phosphate buffer (0.2 M, pH 8.0) was added. After the addition of 1 mL of fluorescamine solution (Sigma, 1 mM in acetone), the fluorescence spectrum was measured by excitation at 366 nm. The emission intensity at 480 nm was taken as a data point. A calibration line was prepared accordingly by using 100 µL aliquots of differently concentrated solutions of APTMS in 30 mL of 0.02 M aqueous NaOH (containing 15 mg of the respective dissolved parent silica) [35]. Repeated analysis of the same sample gave an average relative error of 8%.

### 3.5. Physical Measurements

Nitrogen sorption isotherms were collected at 77 K using a Quantachrome NOVA 2200. Samples were vacuum-degassed at 80 °C for 3 h. The total surface area S_BET_ was calculated by the BET method [36], whereas the external surface area S_Ext_ and the primary mesopore volume V_P_ were determined from the high-pressure linear part of the α_S_-plot [37]. Pore size distributions were calculated from the desorption branches of the nitrogen isotherms using the BJH method [38]. The total pore volume V_tot_ was estimated from the amount of nitrogen adsorbed at a relative pressure of 0.95. A Perkin-Elmer LS50B spectrofluorometer was used for the fluorescamine assays and UV-Vis spectra were measured with a Cary 1E spectrophotometer. Powder diffraction patterns were collected on a STOE StadiP diffractometer operating with monochromatized Cu Kα_1_ radiation.

## 4. Conclusions

The influence of the structural properties of mesoporous silica hosts on the adsorption of guest molecules strongly depends on the specific host-guest interactions. In the case of covalent interactions, the amount of adsorbed molecules is mainly determined by the accessibility of the adsorption sites. Host materials with large pore sizes and multi-dimensional channel systems are advantageous for maximizing the amount of adsorbed guests.

In the case of non-covalent interactions, the spacing or density of the adsorption sites, as well as confinement effects have to be taken into account in addition to the accessibility. Finding a balance between the accessibility of the adsorption sites and confinement is crucial for the synthesis of host-guest systems based on non-covalent interactions. A high accessibility facilitates the inclusion of guest species, but the subsequent lack of confinement causes high leaching rates. Low accessibility can similarly lead to high leaching rates, as guest molecules are preferentially adsorbed at sites on the external surface and on the pore surface close to the pore entrances.
